# Do Anatomical Variations of Sphenoid Sinus Influence Sella Exposure and Residual Disease in Pituitary Surgery? — A Study in an Indian Population

**DOI:** 10.1055/s-0044-1788313

**Published:** 2024-10-25

**Authors:** Aparna Gopalakrishnan, Sivaraman Ganesan, Andi Sadayandi Ramesh, Ananthakrishnan Ramesh, Lokesh Kumar Penubarathi, Kalaiarasi Raja, Jijitha Lakshmanan, Akshat Khushwaha, Koshika Kaushal, Arun Alexander

**Affiliations:** 1 Department of ENT, Jawaharlal Institute of Postgraduate Medical Education and Research (JIPMER), Puducherry, Tamil Nadu, India; 2 Department of Neurosurgery, Jawaharlal Institute of Postgraduate Medical Education and Research (JIPMER), Puducherry, Tamil Nadu, India; 3 Department of Radiodiagnosis, Jawaharlal Institute of Postgraduate Medical Education and Research (JIPMER), Puducherry, Tamil Nadu, India

**Keywords:** sella turcica, sphenoid sinus, pituitary neoplasms, carotid artery, endoscopes, tumor burden

## Abstract

**Introduction** Endoscopic transsphenoidal surgery (ETS) is the standard practice in pituitary surgeries. The sellar exposure becomes the main factor which determines the residual disease in ETS. Not many studies can be found in the literature on the influence of anatomical variations of the sphenoid on intraoperative sella exposure.

**Objective** The aim of the current study is to ascertain whether sphenoid sinus variations play a role in sellar exposure and residual tumor volume.

**Methods** This is a prospective study conducted in a south Indian tertiary care center between June 2020 to June 2022, with 21 study participants who were scheduled to have ETS. The relation of preoperative computed tomography (CT) and magnetic resonance imaging (MRI) parameters with the intraoperative area of sellar exposure and residual tumor volume was evaluated.

**Results** Sphenoid sinus dimensions, like presellar width (mean = 1.89 ± 0.51 cm), maximum width (mean = 2.94 ± 1.09 cm), presellar depth (mean = 1.14 ± 0.55 cm), suprasellar depth (mean = 1.08 ± 0.24 cm), infrasellar depth (mean = 2.36 ± 0.92 cm), presellar height (mean = 2.22 ± 0.47 cm), or the 9 internal carotid artery (ICA)-related measures, did not have any correlation with the mean intraoperative area of sellar exposure (0.57 ± 0.28 cm ^2^ ). Also, the adequacy of sellar exposure did not relate to the residual tumor. Preoperative tumor volume was found to be higher (20.2 [55.3–13.2] cm ^3^ ) in patients with residual tumor compared with those with no residual tumor (5.9 [6.8–5.2] cm ^3^ ). Tumor extension had a significant association with the residual tumor volume.

**Conclusion** According to the present study, anatomical variations of the sphenoid sinus do not influence the adequacy of sellar exposure. Further studies need to be undertaken concerning residual tumor volume as well as preoperative tumor volume and extension.

## Introduction

Endoscopic transsphenoidal surgery (ETS) has become the standard practice in transsphenoidal pituitary surgery. The panoramic view, magnification, and dynamic range of movements provided by the ETS make it a more commonly used approach than microscopic transsphenoidal surgery (MTS). A meta-analysis by Li et al. reports a 52% increase in gross tumor resection rate in ETS compared with MTS. 1

In any surgical procedure, complete tumor resection always depends upon the exposure of the surgical field. In the case of ETS, the sellar exposure becomes the main factor which determines the residual disease. There are only a few studies in the literature on the definition and adequacy of sellar exposure. Even fewer studies have been published on the relation between sellar exposure and residual tumor volume or whether sphenoid sinus variations play a role in sphenoid sinus and sellar exposure.

The sellar exposure is always limited by the proximity of neurovascular structures near the sella turcica. Also, the surgeon must use instruments and manipulate the endoscope in a small space. The variations of the sphenoid sinus are diverse, from basic anatomical variations, like pneumatization of the sphenoid, to risky variations, like termination of intersphenoid septum on the carotid canal and carotid canal wall dehiscence, which can be found preoperatively using computed tomography (CT).

Wang et al. defined adequate sellar exposure in MTS using postoperative CT reconstruction and assessing the residual tumor volume using magnetic resonance imaging (MRI). The anatomical findings seen during the repeat surgery in case of residual or recurrent pituitary adenomas were analyzed by Mattozi et al. to classify inadequate sellar exposure. 2 3 As reported in these studies, the preoperative volume and the nature of the tumor can also influence the total tumor resection rate, even in an adequately exposed sella.

The implications of sphenoid anatomical variations on the sellar exposure have not been explored much in the literature. The intercarotid distance, for example, limits the lateral margins of sellar exposure. Injury to the internal carotid artery (ICA), and cerebrospinal fluid (CSF) leak are probable complications as we increase the sellar exposure.

Our study aims to quantitatively assess the anatomical variations of the sphenoid sinus, preoperative tumor volume, and intraoperative sella exposure in ETS and to establish a correlation between them.

## Methods

### Study Participants

A total of 21 patients (13 females and 8 males) aged 21 to 63 years who opted for ETS for pituitary tumor resection in the department of neurosurgery, in a tertiary care institute from June 2020 to June 2022 were recruited for this prospective study according to the inclusion criteria. The inclusion criteria were all adults over 19 years of age, diagnosed with a pituitary tumor, planned for ETS. The exclusion criteria included 1) patients with a history of sinonasal surgery; 2) patients who have sustained trauma to the sinus previously; and 3) patients with sinonasal malignancy.

### Ethics Approval

Ethics approval was obtained from the human ethics committee of the Institute (JIP/IEC/2020/053) on June 26 ^th^ , 2020. Written informed consent was obtained from all study subjects. The study was conducted by the principles of ethical research as per the Declaration of Helsinki, of 1975 and revised in 2008, and the Indian Council of Medical Research Guidelines for Biomedical Research in Human Participants.

### Computed Tomography and MRI

Preoperatively noncontrast CT of sinuses and T1- and T2-weighted brain MRI were obtained from all study participants. Preoperative CT parameters, such as sphenoid sinus dimensions, ICA-related measurements, and variations of neurovascular structures, were measured as described in the literature. 4 5 These measures were taken according to the earlier studies and measured using the Picture Archiving and Communication System (PACS).

**Fig. 1:**
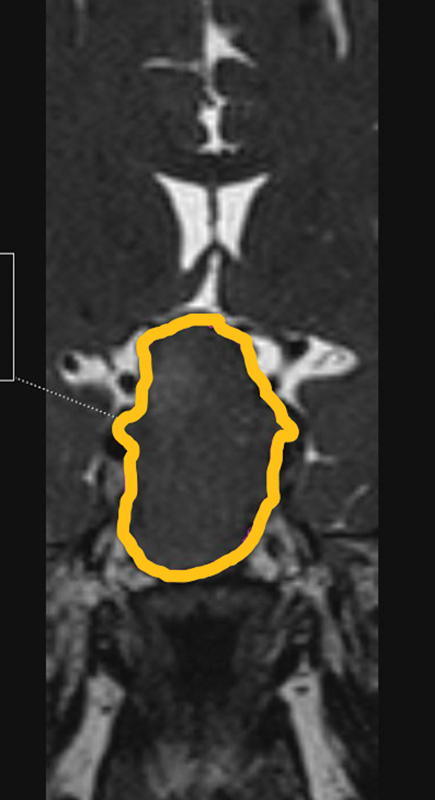
Preoperative tumor volume measured using 3D magnetic resonance imaging (yellow outline).

The preoperative volume of the tumor was measured using 3D MRI in PACS, as shown in Fig. 1 .

According to the tumor extension in MRI, they were classified into grades proposed by the Hardy-Wilson classification system. The tumors within the sella, tumors causing expansion of sella, localized destruction of the sellar floor, and collapse of the sellar floor were graded as I, II, III, and IV, respectively. Wilson classified suprasellar extension of pituitary adenoma as: A - suprasellar extension, B - third-ventricle recess obliteration, and C - abutting and displacing the third ventricle. Classes D and E are reserved for intracranial and parasellar extensions having tumors, respectively. 6

### Surgical Procedure

All 21 patients underwent endoscopic transnasal transsphenoidal surgery (ETTS). Posterior septectomy was done in all patients to allow the binostril approach. Middle turbinectomy, ethmoidectomy, and septoplasty were the procedures done to increase accessibility and reach the sella. The same neurosurgeon and otolaryngologist did all the surgeries.

Videos of all 21 patients undergoing ETS were recorded using AIDA software - Karl Storz AIDA (Advanced Image and Data Acquisition/Archiving System), Tuttlingen, Germany. The sellar exposure was measured using the Image J software (public domain) using the formula (π/4) * b*l, in which b = breadth and l = length or height of the sellar exposure.

### Postoperative Evaluations

After 3 months, a brain MRI was taken to reassess the residual tumor. The tumor volume in the postoperative MRI was compared with the preoperative volume. Total resection was considered when there was no residual tumor in the postoperative MRI. When there was less than 10% residual tumor volume, it was considered partial resection, and more than 10% was considered subtotal resection.

### Statistical Analysis

Data analysis was done using version the SPSS Statistics for Windows (SPSS Inc., Chicago, IL, USA) version 17.0 software. Categorical independent variables such as gender, diagnosis, and symptoms were summarized as frequency and proportions. Continuous variables were summarized as mean ± standard deviation (SD) or median and interquartile range (IQR) based on the normality of data distribution. For linear correlation of two data, the Pearson or Spearman correlation coefficient was used according to the normality of the data. To identify differences between the two groups, the Mann-Whitney U test was used. A *p* -value < 0.05 was considered significant for all statistical tests.

## Results

### Patient Characteristics

Twenty-one patients aged from 19 to 63 years were recruited for this study. The mean age was 42.90 ± 13.01. There were 13 female and 8 male patients. Among the 21 patients who underwent ETS, 8 had nonfunctioning pituitary macroadenoma, and 6 had functioning pituitary adenoma. There were five patients with pituitary microadenoma, one had a diagnosis of Rathke cleft, and another had craniopharyngioma.

Most patients came with initial complaints of diminution of visual acuity and visual field defects (13). Furthermore, four patients had acromegaly, two had Cushing disease, one had infertility, and one had memory loss.

### Computed Tomography Parameters and Area of Sellar Exposure

**Fig. 2:**
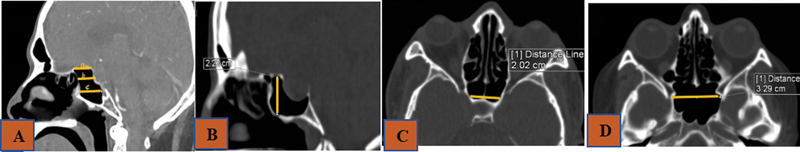
Sagittal computed tomography (CT) view showing ( **A** ); suprasellar depth (a), presellar depth (b), infrasellar depth (c), ( **B** ) Presellar height, axial view CT scan showing ( **C** ) presellar width, and ( **D** ) maximum width.

The geometric measurements of the sphenoid sinus were taken in 17 out of 21 patients. The remaining 4 patients had an expansion of sella and tumor occupying the sphenoid sinus. In seven cases, sphenoid sinus dimensions were measured. In the axial view, presellar width and the maximum width of the sphenoid sinus were measured. In the sagittal view, (i) suprasellar depth was measured at the roof of the sphenoid sinus, (ii) presellar depth was measured at the midpoint of the anterior face of the sella, (iii) infrasellar depth was measured below the sella turcica, and (iv) presellar height was measured from the roof of the sphenoid sinus to the floor perpendicular to the planum sphenoidale ( Fig. 2 ). 7 The mean of these measures is given in Table 1 . These measures were correlated with the mean area of sellar exposure in 17 patients, which came to be 0.57 ± 0.26 cm ^2^ . No statistically significant correlation could be attributed with any of the parameters.

**Table 1:** Sphenoid sinus dimensions and parameters in relation to the ICA

**Parameter**	**mean ± SD (cm) (** ***n*** ** = 17)**
Presellar depth	1.14 ± 0.55
Suprasellar depth	1.08 ± 0.24
Infrasellar depth	2.36 ± 0.92
Presellar width	1.89 ± 0.51
Maximum width	2.94 ± 1.09
Presellar height	2.22 ± 0.47
**ICA parameters**	**mean ± SD (cm) (** ***n*** ** = 21)**
D1	8.85 ± 0.70
D2R	0.18 (0.07–0.25) ^Ω^
D2L	0.10 (0.05–0.16) ^Ω^
D3R	9.78 ± 0.56
D3L	9.74 ± 0.61
D4R	2.63 ± 0.39
D4L	2.60 ± 0.42
D5	1.93 ± 0.32
D6	2.40 ± 0.40
D7	7.47 ± 0.58
A1	12.15 ± 2.4
A2	44.95 ± 9.2

**Abbreviations:** ICA, internal carotid artery; SD, standard deviation.

**Note:** Ω = Median with interquartile range.

**Fig. 3:**
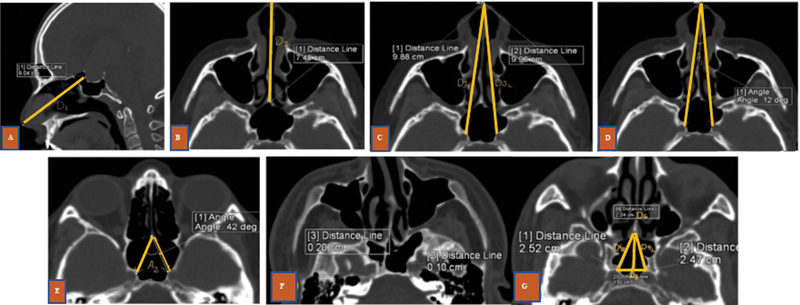
Sagittal window of computed tomography (CT) showing D1 - Distance from the columella to the anterior-thinnest part of sella) ( **A** ), Axial CT view showing ( **B** ) D7 - Distance between the columella of the nose and the anterior wall of the sphenoid, ( **C** ) of D3R and D3L (distance from the columella of the nose to the bony protrusion of carotid), ( **D** ) Axial CT view of A1 (angle between D3R and D3L), ( **E** ) Axial CT view of A2 (angle between D4R and D4L), ( **F** ) D2R and D2L (thickness of the bone between the carotid and the lateral wall of the sphenoid), ( **G** ) D4 - Distance between the anterior wall of the sphenoid and the carotid in the lateral wall of the sphenoid, D5 - Intercarotid distance at the level of the sphenoid sinus, D6 - Measurement of the line bisecting the intercarotid length extending to the anterior wall of the sphenoid.

We measured distance parameters relating to the ICA in an endoscopic approach using the nine parameters ( Fig. 3 ) defined by Feng et al. 4 These measurements were summarized for 21 patients ( Table 1 ), and correlation was done with the mean area of sellar exposure (0.57 ± 0.28). No significant correlation between the area of sellar floor exposure and distance parameters relating to ICA could be found.

Risky anatomic variations like dehiscence and protrusion of the neurovascular structures around the sphenoid were studied. The protrusion was defined as the bulging of a neurovascular structure into the sphenoid sinus with more than half of its circumference inside the cavity 8 and dehiscence, when there is no bony covering or suspiciously thin bony canal wall. 9 In our study, out of 21 patients, 8 had carotid artery protrusion, and 2 had dehiscence. Optic nerve protrusion was seen in three patients and dehiscence in one patient. Vidian canal protrusion was observed in six patients, with all of them showing dehiscence ( Table 2 ). Only one patient had foramen rotundum protrusion, and none had dehiscence. We could not find any association between these variations of neurovascular structures and the area of sellar floor exposure.

**Table 2:** Variations in neurovascular structures and sphenoid sinus

Parameters	Frequency (n) = 21	
	Protrusion	Dehiscence
ICA	8	2
Optic nerve	3	1
Vidian canal	6	6
Foramen rotundum	1	0
Optic nerve type n = 21	Type 1	17
	Type 2	1
	Type 3	1
	Type 4	2
Pterygoid pneumatization n = 21	Type 1	17
	Type 2	2
	Type 3	2
Type of septum n = 17	Absent	1
	Single	9

**Abbreviation:** ICA, internal carotid artery.

Optic nerve types were classified according to Delano classification. Type 1: adjacent to the sphenoid sinus, not causing indentation of the sphenoid sinus wall; type 2: causing indentation on the sphenoid sinus wall; type 3: traversing through the sphenoid sinus; and type 4: traversing adjacent to the sphenoid sinus and posterior ethmoidal cell. Out of 21 patients, 17 had type 1 optic nerve, two had type 4, one had type 2, and another had type 3. Pterygoid pneumatization was also classified into 3 types: most of the patients (17) had type 1 pneumatization, whereas 2 patients had type 2, and the remaining 2 patients had type 3 and 4, respectively. The sphenoid septum was classified according to the termination and type. We could measure these two parameters only in 17 patients due to the tumor expanding the sella and occupying the sphenoid sinus, as discussed earlier. Eleven patients had sellar termination of the septum, five patients had septa of the sphenoid septum ending on the carotid canal, and one had lateral termination of the septum. A single sphenoid septum was found in 9 patients, double in 5 patients, diverging types of the sphenoid septum in 2 patients, and one did not have a sphenoid septum.

The median preoperative tumor volume measured using 3D MRI was 7.36 (20.2–5.9) cm ^3^ ( Table 3 ). The residual tumor was assessed using an MRI after 3 months of surgery. The mean tumor volume in patients with no residual tumor (11) was found to be 6.16 ± 1.95, and those with residual tumor (10) had a median volume of 20.22 (55.55–13.29). In the residual tumor group, the minimum tumor volume was 6.46 cm ^3^ , and the maximum was 57.16 cm ^3^ . There was a significant association between preoperative tumor volume and residual disease ( *p* < 0.001). According to the Hardy-Wilson classification, 10 patients had a suprasellar extension of grade B and above, and all of them were found to have residual disease in the postoperative MRI. It can be inferred from the data that parasellar (grade E), intracranial (grade D), and grade-B or -C suprasellar extension make it difficult to achieve a complete tumor resection ( Table 4 ).

**Table 3:** Association between sellar exposure, preoperative tumor volume, and residual disease

Variables n = 21	Mean ± SD/ median (IQR)	Residual disease		*p* -value*
		Absent ( *n* = 11)	Present ( *n* = 10)	
Area	0.57 ± 0.28 cm ^2^	0.63 ± 0.30	0.59 ± 0.28	0.833
Preoperative tumor volume	7.36 (20.2–5.9) cm ^3^	6.16 ± 1.95	20.2 (55.3–13.2)	< 0.001

**Abbreviations:** IQR, interquartile range; SD, standard deviation.

**Note:** *Mann-Whitney U test.

**Table 4:** Hardy-Wilson grading of patients with residual disease

Hardy-Wilson grade	Nr. of patients ( *n* = 21)	Residual disease ( *n* = 10)
I	4	0
II	4	0
A	6	3
B and above	7	7

The mean sellar exposure area was 0.57 ± 0.28 cm ^2^ , but it had no association with the residual tumor volume.

## Discussion

The present study evaluated the association of the preoperative CT and MRI parameters with the intraoperative area of sellar exposure. No studies have defined adequate sellar exposure for complete tumor resection in an ETS. Our study showed that sella exposure alone is not associated with the residual tumor volume. According to the Hardy-Wilson classification, we had 13 patients with suprasellar or parasellar extension, as mentioned in Table 4 . Among these, 10 patients had residual disease. They either had grade-B or above suprasellar or parasellar and intracranial extensions. In the present study, it was found that the tumor volume and tumor extension to unresectable areas are the factors that significantly influence the total tumor resection ( *p* < 0.001).

Wang et al. studied the association between the area of sellar exposure and postoperative residual tumor volume in microscopic transsphenoidal surgery. The study used multiplanar reconstruction of postoperative CT to measure sella exposure. An MRI scan of the paranasal sinus was taken within one week of surgery to assess the residual disease. It was found that the ratio of sella exposure to the total tumor area and the extent of the disease played a role in the complete removal of the pituitary adenoma. He had found that sellar exposure alone could not be taken as the determining factor for residual tumor. 2

Out of the 21 study participants, we could measure the dimensions of the sphenoid sinus in only 17 patients. There were four patients with sellar floor destruction, and the entire sphenoid sinus was occupied by the tumor. We measured presellar width, the maximum width of sphenoid sinus, as well as infrasellar, presellar, and suprasellar depths. These measurements were like the values reported in a study by Wiebracht and Zimmer 7 ( Table 5 ), but they did not correlate these dimensions with the intraoperative area of sellar exposure as we did in our research. No significant correlation could be found between the sphenoid sinus dimensions and the area of sellar exposure.

**Table 5:** Comparison of sphenoid sinus dimensions and ICA parameters in the previous studies

**Parameter**	**Present study (** ***n*** ** = 17)** **(mean ± SD) (cm)**	**Wiebracht et al.** ***n*** ** = 90** **(mean ± SD) (cm)**
Presellar depth	1.14 ± 0.55	1.3 ± 0.34
Suprasellar depth	1.08 ± 0.24	1.3 ± 0.47
Infrasellar depth	2.36 ± 0.92	2.6 ± 0.71
Presellar width	1.89 ± 0.51	1.3 ± 0.34
Maximum width	2.94 ± 1.09	3.5 ± 0.98
Presellar height	2.22 ± 0.47	2.3 ± 0.45
**ICA parameters**	**Present study (** ***n*** ** = 21)** **(mean ± SD) (cm)**	**Feng et al.** **Median (IQR)** ***n*** ** = 101 (cm)**
D1	8.85 ± 0.70	8.64 (7.74–9.93)
D2R	0.18 (0.07–0.25) ^Ω^	1.73 (0.8–3.6)
D2L	0.10 (0.05–0.16) ^Ω^	
D3R	9.78 ± 0.56	8.55 (7.25–10.59)
D3L	9.74 ± 0.61	
D4R	2.63 ± 0.39	1.69 (0.81–2.65)
D4L	2.60 ± 0.42	
D5	1.93 ± 0.32	2.16 (1.28–3.01)
D6	2.40 ± 0.40	1.21 (0.39–2.45)
D7	7.47 ± 0.58	7.26 (5.51–8.66)
A1	12.15 ± 2.4	14.9 (10.2–21.8)
A2	44.95 ± 9.2	85.49 (41.3–130)

**Abbreviations:** ICA, internal carotid artery; IQR, interquartile range; SD, standard deviation.

**Note:** Ω Median with interquartile range.

The ICA-related CT measurements were taken, as described in a study by Feng et al. 4 The measurements in the present study were comparable to the values in the study of Feng et al. The only value which differed between the studies was A2, the angle between D4R and D4L. It had an average of 45.92 ± 9.2 degrees in our study, whereas Feng et al. found an average of 85.9, with values ranging from 49.9 to 121 degrees. No correlation could be established between ICA-related CT measurements and the adequacy of sellar exposure.

Risky anatomic variations of neurovascular structures, such as the ICA, optic nerve, maxillary nerve, vidian canal protrusion, and dehiscence, were assessed in the preoperative CT. Among the 21 patients, 13 had ICA bony canal wall protrusion, and 2 had dehiscence of the carotid canal wall. The optic nerve was dehiscent in 1, and protrusion was found in 3 patients. These findings were comparable to those of the study on cadavers by Safarian et al., in which 15% of cadavers had optic nerve dehiscence and 20% had carotid canal wall dehiscence. 10 In our study, 6 patients had vidian canal dehiscence and protrusion, and only one had maxillary nerve protrusion. We had almost the same ratio of these occurrences as that reported in a study by Birsen et al. 9 However, none had any significance in relation to the area of sellar exposure.

We used Delano's 11 classification of optic nerves and Alec Vaezi's 5 classification of pterygoid process pneumatization. Termination of the sphenoid septum can be on the sellar floor, laterally, or on the bony covering of the ICA. Previous studies have found that 14% of the study population have septum ending on the carotid canal. 12 This can cause accidental injury to the ICA if the surgeon is not careful during the removal of intersphenoid sinus septum for sellar exposure. The frequencies of these variations in our study are enumerated in Table 2 . The present study was conducted in a single center and limited by a small sample size.

## Conclusion

The present study could not find any correlation between the geometric measures of the sphenoid sinus, measured using CT, and intraoperative sellar exposure. Due to the limited sample size, further studies with larger cohorts are necessary to validate these findings, especially regarding residual disease as well as preoperative tumor volume and extension.
